# Targeting ferroptosis as a potential strategy to overcome the resistance of cisplatin in oral squamous cell carcinoma

**DOI:** 10.3389/fphar.2024.1402514

**Published:** 2024-04-22

**Authors:** Rongkun Chen, Shuyu Zhu, Ruoyu Zhao, Wang Liu, Luxin Jin, Xiaobin Ren, Hongbing He

**Affiliations:** ^1^ Yunnan Key Laboratory of Stomatology, School of Stomatology, Kunming Medical University, Kunming, China; ^2^ Department of Periodontology, Kunming Medical University School and Hospital of Stomatology, Kunming Medical University, Kunming, China; ^3^ Department of Oral Implantology, Kunming Medical University School and Hospital of Stomatology, Kunming Medical University, Kunming, China

**Keywords:** ferroptosis, OSCC, HNSCC, cisplatin, chemoresistance, chemotherapy

## Abstract

Oral squamous cell carcinoma (OSCC) is a crucial public health problem, accounting for approximately 2% of all cancers globally and 90% of oral malignancies over the world. Unfortunately, despite the achievements in surgery, radiotherapy, and chemotherapy techniques over the past decades, OSCC patients still low 5-year survival rate. Cisplatin, a platinum-containing drug, serves as one of the first-line chemotherapeutic agents of OSCC. However, the resistance to cisplatin significantly limits the clinical practice and is a crucial factor in tumor recurrence and metastasis after conventional treatments. Ferroptosis is an iron-based form of cell death, which is initiated by the intracellular accumulation of lipid peroxidation and reactive oxygen species (ROS). Interestingly, cisplatin-resistant OSCC cells exhibit lower level of ROS and lipid peroxidation compared to sensitive cells. The reduced ferroptosis in cisplatin resistance cells indicates the potential relationship between cisplatin resistance and ferroptosis, which is proved by recent studies showing that in colorectal cancer cells. However, the modulation pathway of ferroptosis reversing cisplatin resistance in OSCC cells still remains unclear. This article aims to concisely summarize the molecular mechanisms and evaluate the relationship between ferroptosis and cisplatin resistance OSCC cells, thereby providing novel strategies for overcoming cisplatin resistance and developing new therapeutic approaches.

## 1 Introduction

Globally, head and neck cancer (HNC) ranks as the sixth most common type of cancer. OSCC is one of the most prevalent forms of the HNC, accounting for approximately 2% of all cancers globally and 90% of oral malignancies, with around 377,000 new cases diagnosed and 177,000 deaths annually (Sung et al., 2021). Worldwide, OSCC is a crucial public health problem, which ranks as the fourth most prevalent type of cancer in low/medium-income countries (Nagao and Warnakulasuriya, 2020). The conventional and standard treatment of OSCC is generally surgery followed by chemotherapy and radiotherapy. As a conventional treatment option for various types of cancer, including OSCC, chemotherapy is also a popular adjunct treatment for the advanced stages (Tan et al., 2023). However, due to the locally advanced stage because of a lack of awareness and a delay in initial diagnosis, OSCC patients still have a 50%–60% 5-year survival rate, despite the achievements in surgery, radiotherapy, and chemotherapy techniques over the past decades (Chinn and Myers, 2015).

Cisplatin, as a classical platinum-based chemotherapeutic agent, was discovered in 1845, which is widely used in solid cancer as a valid treatment choice (Ghosh, 2019). However, the resistance to chemotherapeutic reagents, such as cisplatin, leads to recurrence and metastasis after treatment. Plenty of basic and clinical studies aimed to reveal the mechanisms and overcome drug resistance.

As a novel form of programmed cell death, ferroptosis can be characterized by its morphological features, which was initially reported in 2012 (Dixon Scott et al., 2012). The main morphological appearance of ferroptosis is the alternations in mitochondria, for example, reduction or loss of mitochondrial cristae, rupture of the outer mitochondrial membrane, and increase in mitochondrial membrane density. Besides, neither the alternations in typical necrosis, such as the cellular swelling and the rupture of the cellular membrane, nor the characteristics of apoptosis, like the shrinkage of the nucleus and the condensation of chromatin, cannot be observed in ferroptosis cells. Researchers have discovered that the initiation of ferroptosis is triggered by the build-up of iron-dependent lipid peroxides in cells (Mou et al., 2019). Nowadays, ferroptosis has been discovered to correlate with chemoresistance, and inducing ferroptosis can reverse the drug resistance (Zhang et al., 2022). This review aims to concisely summarize the current molecular mechanisms of ferroptosis and cisplatin resistance in OSCC cells, then discusses how the inducing of ferroptosis can be an underlying strategy to overcome the cisplatin resistance in the realm of chemotherapy and expects that this review could stimulate some novel thoughts of developing ferroptosis-based therapies to reverse cisplatin-resistant in OSCC.

## 2 The mechanisms and pathways of ferroptosis

**FIGURE 1:**
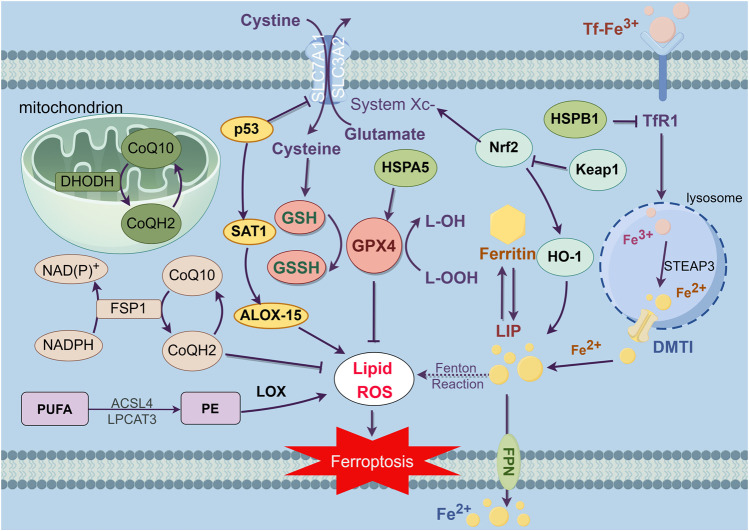
This figure exhibits the main regulation mechanisms of ferroptosis, which can be separated into three pathways. The first part is system XC-/GSH/GPX4 pathway. As an antitransporter, system XC- pumps cystine in the cell while pumping glutamate out, then synthesizes GSH which decreases ROS under the influence of GPX4, thereby inhibiting ferroptosis. Second, the iron metabolism, TfR1 inhales Tf-Fe^3+^ complex, then separating Fe^3+^ and reverting Fe^3+^ to Fe^2+^, which forms the reactive LIP. As the intracellular Fe^2+^ aggregates, resulting in the Fenton reaction, inducing LIP to produce large amounts of lipid peroxides and oxidative free radicals, which triggers ferroptosis. The third mechanism is related to the regulation of lipid metabolism. As a kind of PUFA, PE is synthesized and remodeled through two kinds of essential enzymes, ACSL4 and LPCAT3. The accumulation of PE is the symbol and the destination of ferroptosis. Furthermore, it has been revealed that the NADPH/FSP1/CoQ10 pathway and mitochondria defense system dihydroorotate dehydrogenase (DHODH) are independent parallel systems that cooperate with GPX4 pathway to inhibit ferroptosis.

Ferroptosis can be promoted by iron ions and lipids, such as polyunsaturated fatty acids (PUFA), while can be inhibited by some products of amino acid metabolism, like glutathione (GSH) serving as a substrate of glutathione peroxidase 4 (GPX4), which can relieve the toxicity of lipid peroxides. When cells are unable to eliminate the excessive intracellular reactive oxygen species (ROS), the accumulated lipid peroxidation will induce ferroptosis (Louandre et al., 2013; Hassannia et al., 2019) (Figure 1).

### 2.1 Metabolism of iron ions

Iron ions are key materials in the synthesis of DNA and protein and in the process of cell growth. The lack of iron ions will result in the growth arrest and death of cells (Galaris et al., 2019). Physiologically, iron ions are in the form of Fe^2+^ and Fe^3+^, which can maintain relative homeostasis of redox states and ion concentrations. Extracellularly, Fe^3+^ binds into transferrin (Tf) to form the Tf- Fe^3+^ complex. Then, the Tf- Fe^3+^ complex will be transported to different tissues and organs through blood circulation. Transferrin receptor 1 (TfR1) in the cell membrane can recognize Tf- Fe^3+^ complex and inhale the complex into the cell by endocytosis, then separate Fe^3+^ from the complex in the lysosome. After that, the Fe^3+^ is reduced to Fe^2+^ in the lysosome by six transmembrane epithelial antigen of the prostate 3 (STEAP3). Subsequently, Fe^2+^ will be transferred to cytoplasm by divalent metal transporter 1 (DMT1) (Sendamarai et al., 2008; Wolff et al., 2018). The free Fe^2+^ in the cytoplasm will form the reactive labile iron pool (LIP). Fe^2+^ in LIP can be transferred to other organelles, and then to be participated in the activation of protein activity in these organelles (Mu et al., 2021). Ferroportin (FPN) can export the Fe^2+^ to the outside of the cells, thus avoiding the accumulation of excess iron ions (Yang et al., 2014). Normally, LIP would maintain the balance of iron ions intracellular, while pathologically, intracellular Fe^2+^ aggregates, resulting in the Fenton reaction, which can produce large amounts of lipid peroxides and oxidative free radicals. Failing to clear the excess free radicals will trigger ferroptosis (Song et al., 2019; Mu et al., 2021).

Nuclear factor erythroid 2-related factor 2 (Nrf2) is a major intracellular modulator of the antioxidant defense system, which is involved in signal transduction related to multiple intracellular defense mechanisms. Physiologically, Nrf2 acts as a compound with Kelch-like ECH-associated protein 1 (Keap1) in the cellular. When being stimulated by oxidative stress, excess ROS will dissociate Nrf2 from the compound and activate a series of antioxidant stress factors (Korytina et al., 2019). HO-1 is a target protein downstream of Nrf2, which can degrade heme to release biliverdin, CO, and Fe^2+^. On one hand, the activation of HO-1 would lead to the accumulation of Fe^2+^ which can induce ferroptosis. On the other hand, Fe^2+^ could be oxidized to Fe^3+^, then it will be stored by ferritin or exported outside the cell by FPN-1, which has been proven to contribute to inhibit ferroptosis (Fahrer et al., 2023), suggesting that the role of HO-1 in ferroptosis may depend on different conditions and need further research. In conclusion, the excess iron ions are necessary for the occurrence of ferroptosis. When intracellular iron-related proteins and iron homeostasis are dysregulated, intracellular iron ion concentration will increase, inducing the accumulation of an excess of ROS, and resulting in ferroptosis.

### 2.2 Metabolism of lipids

Ferroptosis is marked by the build-up of lipid peroxides that rely on iron for their formation. Lipid peroxides are a kind of intracellular ROS. PUFA and monounsaturated fatty acid (MUFA) are two kinds of the reaction substrates of lipid peroxidation. Between them, PUFA is more susceptible to be oxidized than MUFA (Yang et al., 2016). The free PUFA undergoes esterification to become membrane phospholipids, which can induce ferroptosis after further oxidation (Kagan et al., 2017). The symbol and the destination of ferroptosis is the accumulation of PUFA in cells (D Herde and Krysko, 2017). The difference in PUFA levels would cause the different rates of intracellular lipid peroxidation, and the varied intensity of ferroptosis. ROS is a general term for peroxides with oxygenated radicals in organisms, which can destroy intercellular structures such as RNA, DNA and lead to cell death (Lin et al., 2018). As a group of PUFA-related phospholipids, phosphatidyl ethanolamine (PE) is rich in arachidonic acid which has been proven to be the key material in activating ferroptosis (Kagan et al., 2017). Intercellular acyl-CoA synthetase long-chain family member 4 (ACSL4) and lysophosphatidylcholine acyltransferase 3 (LPCAT3) are essential enzymes in the process of lipid peroxidation. ACSL4 is involved in the synthesis of PE, while LPCAT3 contributes to the remodeling of PE. The lack of these two kinds of enzymes results in a PUFA decrease following the reduction in PE, which would then suppress the process of ferroptosis (Doll et al., 2017; Kagan et al., 2017). The peroxidation of PUFA is also catalyzed by lipoxygenase (LOX). When reducing the amount of intracellular LOX can inhibit the accumulation of PUFAs, which results in the inhibition of ferroptosis. In a word, dysregulation of lipid peroxide metabolism in cells is the premise for the occurrence of ferroptosis.

### 2.3 Metabolism of amino acids

System Xc-, composed of SLC7A11 and SLC3A2, is a crucial intracellular antioxidant protein, which is widely dispersed in phospholipid bilayers. It can transport amino acids at a 1:1 ratio, pumping cystine into the cell while pumping glutamate out of the cell, thereby maintaining a dynamic balance between these two amino acids in cells (Dixon Scott et al., 2012). Intracellularly, cystine is converted to cysteine catalyzed by enzymes, then combining with glutamic acid and glycine to generate GSH (Koppula et al., 2020). As the predominant intracellular antioxidant, GSH plays a crucial role in reducing ROS under the influence of GPXs, thereby safeguarding cells against oxidative harm. In the GPX family, GPX4 inhibits the accumulation of lipid peroxides and is a pivotal inhibitor of ferroptosis. With a strong antioxidant capacity, GSH can be oxidize to glutathione disulfide (GSSG) by GPX4, and then reduce lipid peroxides to the corresponding alcohols. Normally, cancer cells have a higher level of ROS than non-cancer cells, while reducing the level of ROS inhibits the proliferation of cancer cells. However, as the level of ROS increased, cancer cells are facing a higher burden on antioxidant systems, which leading to an overexpression of GSH or Nrf2 in plenty of cancer cells (Kennedy et al., 2020). Nrf2-Keap1 can also upregulate system Xc- and increase the secretion of glutamate thereby inhibiting ferroptosis (Fan et al., 2017). RSL3 and erastin can respectively act on GPX4 and SLC7A11, then degrading the antioxidant ability of the cells and leading to the accumulation of ROS, eventually resulting in cell ferroptosis. As the latest reported ferroptosis agonist, Fin56 can exert synergy effects with the activation of cytogenesis autophagy to promote the degradation of GPX4, though its mechanism needs further research (Sun et al., 2021). System Xc-/GSH/GPX4 is a pivotal inhibitory pathway of ferroptosis (Dixon Scott et al., 2012).

### 2.4 Other factors

Furthermore, ferroptosis is also modulated by several factors, including ferroptosis suppressor protein 1 (FSP1), p53, heat shock protein (HSP), and mitochondria

*p53* is an important antioncogene, which can regulate cancer cell proliferation, differentiation, and death. Recent studies have found that *p53* can combine with the SLC7A11 promoter leading to inhibit the expression and the transport capacity of the system Xc-, which results in the decline in cellular antioxidant ability and the build-up of lipid peroxide, and finally induces ferroptosis (Jiang et al., 2015a; Jiang et al., 2015b). Additionally, abundant *p53* can activate spermidine/spermine N1‐acetyl‐transferase 1 (SAT1), which can regulate the arachidonate 15‐lipoxygenase (ALOX-15) resulting in the increase of intracellular lipid peroxidation, and will eventually induce ferroptosis (Ou et al., 2016).

FSP1 is a novel important ferroptosis-regulating protein that can restrain cells from ferroptosis when GPX4 pathway is blocked. As a kind of oxidoreductase of NADPH-dependent Coenzyme Q (CoQ), FSP1 can reduce coenzyme Q10 (CoQ10) to dihydro-ubiquinone (CoQH_2_). CoQH_2_ is a kind of antioxidant that traps lipophilic free radicals, which inhibits the build-up of lipid peroxides (Bersuker et al., 2019; Doll et al., 2019). As a parallel system, the NADPH/FSP1/CoQ10 pathway can not only work cooperate with the GPX4 pathway, but also in the absence of the GPX4 pathway to inhibit ferroptosis.

HSP is a kind of highly conserved molecular chaperone, which will be expressed under environmental pressure, to protect cells from different types of cell death. Heat shock protein beta-1 (HSPB1) can decrease cellular iron uptake, reducing intracellular ROS and inhibiting ferroptosis (Sun et al., 2015). Heat shock protein family A member 5 (HSPA5) can combine with GPX4, thereby keeping the stability of GPX4 from GPX4 protein degradation, thus inhibiting cell lipid peroxidation and ferroptosis (Zhu et al., 2017).

The defense system localized at mitochondria is mediated by dihydroorotate dehydrogenase (DHODH) which is an enzyme crucial for pyrimidine synthesis reducing CoQ10 to CoQH_2_ at the inner mitochondrial membrane, thereby limiting the buildup of lipid peroxides. Notably, when the expression of GPX4 is downregulated, flux through DHODH will increase significantly, leading to increased expression of CoQH_2_, thereby protecting cells from lipid peroxidation and inhibiting ferroptosis in mitochondria. Emerging proofs suggests that the potential role of targeting DHODH to induce mitochondria damage and the downregulation of both mitochondrial GPX4 and DHODH will lead to mitochondrial lipid peroxidation, ultimately triggering ferroptosis (Mao et al., 2021).

## 3 Relationship between ferroptosis and cancer cells

Cancer cells are cells that have lost normal physiological regulation and developed metabolic plasticity which has unlimited potential for growth. To meet the demands of their constant growth, malignant cells have a higher appetite for iron than normal cells (Hassannia et al., 2019). The specific metabolic reprogramming observed in cancer cells has been linked to an increased susceptibility to ferroptosis (Friedmann et al., 2019). As the iron ions accumulates in cancer cells, it will lead to an elevated level of lipid peroxides and ROS, while downregulating the level of ROS inhibits the proliferation of cancer cells. To cope with this characteristic, the expression of GSH or Nrf2 in malignant cells is upregulated to maintain a balanced level of ROS, which helps them to avoid ferroptosis, but it also results in the ferroptosis vulnerability of cancer cells will increase significantly when the GSH-GPX4 pathway is blocked (Kennedy et al., 2020).

### 3.1 The “iron addiction” of cancer cells

The high-speed proliferation characteristic of cancer cells results in a higher requirement of iron in these cells than in normal cells. As the most popular way for cancer cells to obtain iron, TfR1 is highly expressed in many cancer cells. Normally, the high expression of TfR1 indicates an advanced stage and poor prognosis of tumors (Candelaria et al., 2021).

A previous study has found that various cancer cells are sensitive to ferroptosis inducers (Wu Y. et al., 2023a; Feng et al., 2023; Shan et al., 2023; Zhang et al., 2023). Cancer stem cells (CSCs) are unique cancer cell subpopulations which have stem cell-like characteristics, the capacity for self-renewal and multidirectional differentiation allowing them to promote tumor growth and heterogeneity which is also a key reason for tumor recurrence and metastasis (Marquardt et al., 2018). However, CSCs can be induced death selectively through ferroptosis inducers by increasing sensitivity of cancer cells to chemotherapy, thereby eliminating malignant cells (Walcher et al., 2020). In tumor microenvironment, CSCs have a much higher efficiency of iron absorption than non-CSCs, with an increased expression level of Tf and TfR1, indicating that Tf and TfR1 are essential for the survival of CSCs, and emphasizing the critical importance of iron within these subgroups (Schonberg et al., 2015; Hamaï et al., 2017). The synthesis of polyunsaturated fatty acid-containing phospholipid (PUFA-PL) is significantly enhanced in CSCs, forcing CSCs to rely on GPX4 to detoxify lipid peroxides, indicating that CSCs are extremely vulnerable to ferroptosis when the pathway is being inhibited (Viswanathan et al., 2017; Bersuker et al., 2019; Doll et al., 2019).

Glioblastoma (GBM) is a highly malignant brain cancer with a poor treatment effect and prognosis. Researchers have found that GBM cells request more iron than normal brain cells, which upregulates the expression of TfR1 to intake more Tf- Fe^3+^ complexes to achieve more iron ions (Schonberg et al., 2015; Kawak et al., 2023). As a malignant cancer which occurs in the ovarian epithelium, the high-malignant serous ovarian cancer (SOC) has a higher expression of TfR1 and a lower one of FPN than the low-malignant SOC (Wu et al., 2022). All these studies manifest that cancer cells are addicted to iron.

### 3.2 The “vulnerability” of cancer cells to ferroptosis

The development of resistance of malignant cells to chemotherapy leads to recurrence and metastasis of tumor after treatment. Genes carried by these cancer cells may have been mutated which helps tumors to escape from cancer treatment (Shen et al., 2020). Moreover, metabolic and epigenetic plasticity can be altered by malignant cells to develop resistance to chemotherapy. This adaptability allows them to survive and continue to proliferate even after treatment, which brings heterogeneity to cancer cells, allowing them to be more resistant to conventional therapy strategies (Marine et al., 2020). As the research continues, the vulnerability of cancer cells to ferroptosis has been discovered. This vulnerability is mostly manifested in the dependence of cancer cells on iron, as well as the regulation of the epigenetic plasticity of iron in the drug-resistant status of cancer cells.

Studies have revealed that autophagic degradation of ferritin results in the release of unstable iron, which would lead to cellular ferroptosis (Mancias et al., 2014). In the pancreatic ductal adenocarcinoma mouse model, cysteine depletion also causes cellular ferroptosis (Badgley et al., 2020). The maintenance of resistance to chemotherapy of cancer cells highly depends on the pathway of lipid peroxidase, which reveals that the main characteristic of the mesenchymal status of cancer cells is the enrichment and esterification of PUFAs on their membranes (Hangauer et al., 2017). These PUFAs induce cellular ferroptosis when being catalyzed by LOX. Astrocytes are dependent on the presence of PUFA-rich membranes which can significantly alter cellular metabolic activity, manifesting that cells are forced to reprogram neurons, resulting in enhanced vulnerability of ferroptosis in cells (Gascon et al., 2016). As a kind of enzyme involved in the synthesis of PE, the level of ACSL4 is reduced in imatinib-resistant gastrointestinal stromal tumors cells, while upregulating the expression ACSL4 can induce ferroptosis and alleviate the resistance of imatinib (Cui et al., 2024). It is shown that the expression of Nrf2 is increased in plenty of cancer cell. Drug-resistant malignant cells can even inhibit ferroptosis through upregulating the level of Nrf2 (Adinolfi et al., 2023). Arenobufagin, a bioactive component extracted from toads, can reduce the expression of Nrf2 in colorectal cancer and gastric cancer, inducing ferroptosis, thereby inhibiting proliferation, migration and invasion of malignant cells. Nevertheless, activation of Nrf2 can inhibit ferroptosis and reverse these effects (Long et al., 2024; Wang et al., 2024).

Lipid peroxides are unstable substrates, which can easily be converted to reactive oxygen radicals when being catalyzed by iron ions, then forming toxic lipid peroxidation catabolic products, such as phospholipid hydroperoxides, etc (Yang et al., 2016). If these lipid peroxidation catabolic products are not degraded by ferroptosis-inhibiting systems such as GPX4 or FSP1, they will eventually lead to cellular ferroptosis (Kagan et al., 2017). This vulnerability is manifested in different drug-resistant cancer cells, including *ZEB1*-driven cells which are susceptible to epithelial-mesenchymal transition (EMT) (Krebs et al., 2017). It is shown that the overexpression of *ZEb1* increases the synthetic levels of PUFAs, which upregulates the levels of active lipid peroxides, enhancing the sensitivity of EMT cells to ferroptosis (Han et al., 2021). Through differentially expressed genes (DEGs), it is shown that palmitoyl protein thioesterase 1 (PPT1) is a core gene in OSCC. Overexpression of PPT1 enhances the proliferation of OSCC cells and associates with a poor prognosis, by upregulating the level of GPX4 and inhibiting ferroptosis, while knockdown PPT1 can reverse these effect and increase the sensitivity to ferroptosis inducers (Luo et al., 2024). As the inducers of ferroptosis, RSL3 and erastin have been proved to inhibit the system Xc-/GSH/GPX4 pathway, which provides novel ideas for treating drug-resistant cancer cells.

## 4 Mechanism of cisplatin resistance in OSCC cells

**FIGURE 2:**
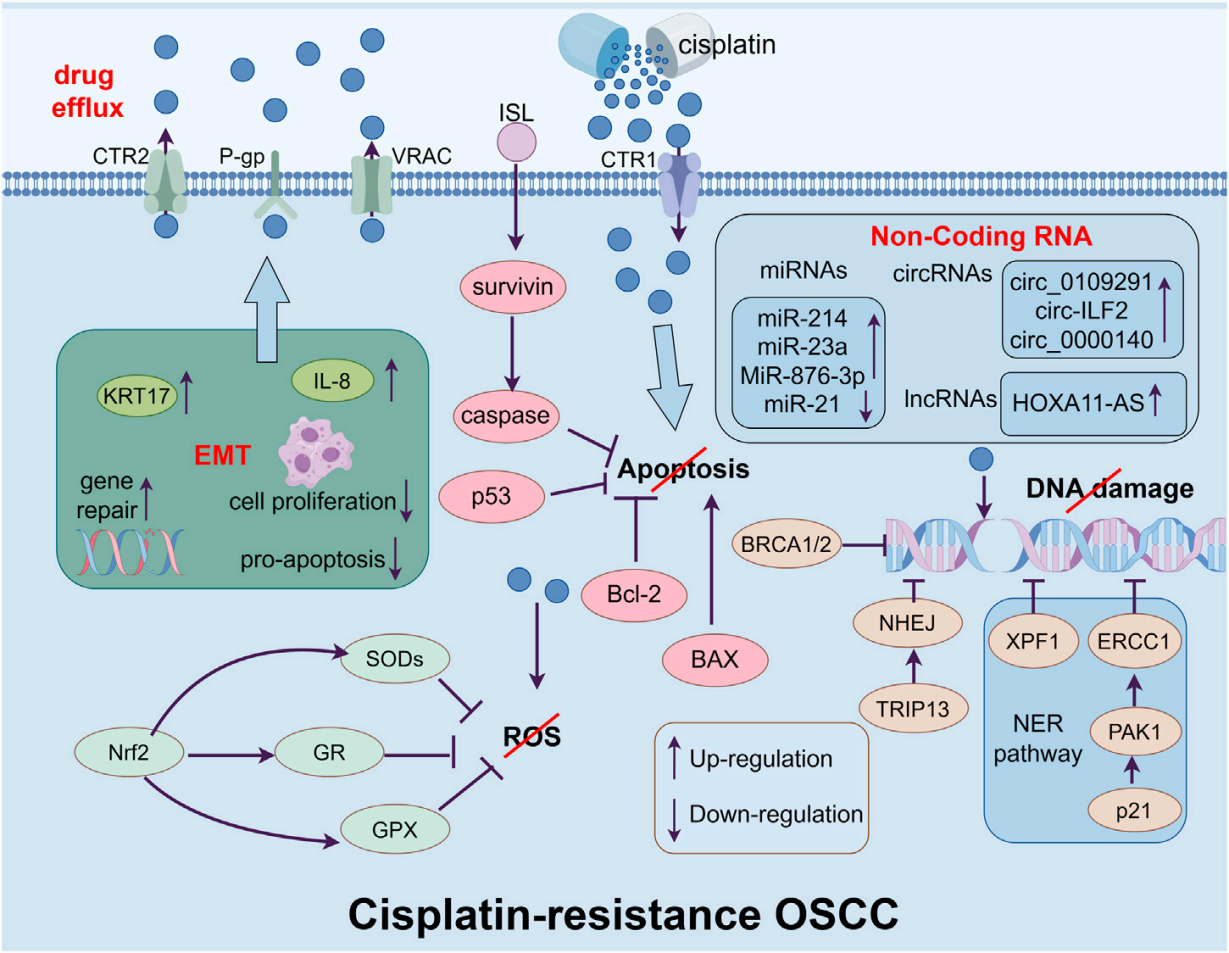
The figure shows the main mechanisms of cisplatin-resistance in OSCC cells, which include enhancing drug efflux ability form drug transporters, inhibiting apoptosis, repairing the damage of DNA causing by cisplatin, limiting the accumulation of ROS in cytoplasm, inducing EMT and regulating the level of non-coding RNA.

As the most commonly utilized chemotherapy medication, cisplatin has a high antitumor activity against many cancers and serves as the first-line treatment for locally advanced OSCC. The absorption of cisplatin is mediated by copper transporter protein 1 (CTR1). Those cells with higher expression of CTR1 have a higher level of cisplatin, which gives them a better expectation of chemotherapy (Ghosh, 2019). Once the cisplatin reaches the cytoplasm, it will be activated by replacing the chlorine atoms with water molecules (Dasari and Tchounwou, 2014). Then it will target to make lesions in DNA by producing oxidative stress such as ROS which leads to peroxidation of lipids and depletion of sulfhydryl groups. If the amount of damaged DNA is beyond the capacity of the DNA repair system, it will eventually lead to cell death through apoptosis (Galluzzi et al., 2012). However, cancer cells become resistant to chemotherapy medications such as cisplatin after being exposed to it several times, leading to recurrent and metastasis which greatly limits the clinical practice of it (Sha et al., 2021). Thus, it will be beneficial to provide potential therapeutic strategies by understanding the mechanism of cisplatin resistance in OSCC (Figure 2).

### 4.1 Exceeding export of cisplatin

ATP-binding cassette transporter (ABC transporter) is a kind of membrane protein which exports drugs and toxins from the cell (Fletcher et al., 2016). As the first found ABC transporter, in higher expressed OSCC cells, P-glycoprotein (P-gp) can export cisplatin thereby inhibiting its sensitivity (Kim et al., 2016a). Additionally, the export of cisplatin is also mediated by copper transporter protein 2 (CTR2). It is shown that the knockdown of CTR2 greatly increases the level of cisplatin in tumor cells, thereby significantly enhancing its drug effect (Blair et al., 2011). The patients with a higher level of CTR2 have a poorer prognosis in cisplatin-based chemotherapy. Moreover, volume-regulated anion channel (VRAC) is another kind of transmembrane protein which can export ions and other osmotic agents when the cell is swelling (Jentsch et al., 2016). The deletion of VRAC drastically enhances the resistance of cancer cells to cisplatin (Siemer et al., 2021).

### 4.2 Enhanced DNA repair mechanism

Once the cisplatin enters the cells, it will induce DNA damage and oxidative stress. In response to this damage, cells enhance the DNA repair system to maintain genetic integrity, which causes drug resistance. As an important way to repair DNA, the nucleotide excision repair pathway (NER pathway) can moderate the sensitivity of platinum-based chemotherapy drugs (Marteijn et al., 2014). Excision repair cross-complementing 1 (ERCC1) and Xeroderma pigmentosum complementation group F protein 1 (XPF1) are two kinds of pivotal endonucleases in the NER pathway. These two enzymes, acting as the step that controls the pace of the NER process, can associate with each other to form a compound that facilitates the elimination of platinum adducts (Sabatella et al., 2021). The overexpression of *p21* protein activates kinase 1 (PAK1) to upregulate the level of ERCC1 in OSCC cells, which desensitizes the cancer cells to cisplatin (Lin et al., 2017). The overexpression of ERCC1 and XPF1 patients normally leads to a shorter progression-free survival and overall survival (Chiu et al., 2011; Zuo et al., 2023). The homologous recombination pathway (HR) which is mediated by *BRCA1/2* will continue to repair them when the NER pathway is unable to repair the DNA lesions (Murai and Pommier, 2023). Patients with a lower level of *BRCA1* have a longer overall survival (Burcher et al., 2021). As another DNA repair system, non-homologous end joining (NHEJ) can also induce cisplatin resistance through the overexpression of thyroid hormone receptor interactor 13 (TRIP13) (Banerjee et al., 2014).

### 4.3 Detoxification of reactive oxygen species

Over the past decades, cisplatin-based cell death was thought to be caused by DNA damage. However, it has been shown that the vital role of the accumulation of oxidative stress, such as ROS, in cisplatin-induced cell death (Kuo et al., 2022). Cisplatin can induce mitochondria to release ROS into the cytoplasm. As the ROS accumulates, the high level of oxidative stress contributes to lesions of lipids, proteins, and DNA which can induce cell death (Ma et al., 2016). When facing the overexpressed ROS, cells develop an intricate defending system consisting of superoxide dismutase (SOD), GPX, and glutathione reductase (GR), which can be induced by Nrf2, thereby reversing the accumulation of ROS and prevent cell death (Noman et al., 2020). Researchers have discovered that cisplatin-resistant OSCC cells have a high expression of Nrf2 (Zeng et al., 2021; Praharaj et al., 2023). As a kind of natural flavonoid, wogonin can selectively kill cancer cells by inducing the accumulation of intracellular ROS and decreasing the expression of Nrf2 to reverse the cisplatin resistance (Kim et al., 2016b). It is shown that in ovarian cancer, the expression of deoxycytidine triphosphate pyrophosphatase 1 (DCTPP1) increases after treating with cisplatin, which associates with a poor prognosis, especially in cisplatin-resistant cells. Downregulating the level of DCTPP1 suppresses the proliferation of cancer cells, leads to a higher level of intracellular ROS, and increases the sensitivity to cisplatin, indicating the crucial rule of ROS in cisplatin treatment (Wang et al., 2022).

### 4.4 Blocking of the apoptosis pathway

Platinum-based chemotherapy drugs can induce cell death through apoptosis. The cisplatin resistance is correlated with genes and proteins associated with apoptosis dysregulation (Siu et al., 2019; Zhang et al., 2019). B-cell lymphoma 2 (Bcl-2) is a kind of apoptosis-regulating proteins, which consists of anti-apoptosis protein and pro-apoptosis protein (Kaloni et al., 2023). Bcl-2 is overexpressed in drug-resistance OSCC cells and the knockdown of it can reverse the resistance to cisplatin (Xiong et al., 2016; Hsu et al., 2019; Qiao et al., 2021). Moreover, as a member of the inhibitor of apoptosis proteins (IAPs), survivin can regulate G2/M cell cycle transition and bind with caspase to inhibit cancer cells from apoptosis, resulting in resistance to chemotherapy, including cisplatin resistance (Ju et al., 2018; Kondapuram et al., 2023). Isoliquiritigenin (ISL) is a natural compound which can suppress survivin from phosphorylation and facilitate survivin for ubiquitination degradation. Combined with cisplatin, ISL can overcome cisplatin resistance caused by the overexpression of survivin in OSCC cells (Zhou et al., 2023). As an important antioncogene, *p53* can also trigger apoptosis in cells (Aubrey et al., 2018). The acetylation and transactivation of *p53* can significantly enhance the sensitivity of apoptosis induced by cisplatin in OSCC cells (Li et al., 2020), while the mutation of *p53* leads to cisplatin resistance in OSCC cells (Alam et al., 2019).

### 4.5 Enhanced EMT capacity

As a cellular reprogramming event, EMT is a process that epithelial cells undergo transformation into mesenchymal cells, which allows cancer cells to invade, extravasate, and, eventually, form metastases (Wangmo et al., 2020). Researchers have found that EMT is likely to be a partial and flexible reprogramming progress in cancer cells that enables them to express mesenchymal and epithelial genes simultaneously (Yang et al., 2020). Recently, EMT has been deemed to be linked with CSC phenotype and drug resistance (Dongre and Weinberg, 2019). EMT cells have increased the expression of gene repairing mechanisms, drug efflux, inhibited cell proliferation, and pro-apoptotic pathways (Shibue and Weinberg, 2017). The level of genes alters based on the requirement of the needs of cancer cells (Pastushenko et al., 2018). It has been shown that the expression of transcription factors like *Snail*, *Twist1* and *Zeb1* are increased in EMT cell, which promote cells resistant to chemotherapy, making them crucial targeting points for determining sensitivity or resistance for cancer cells to chemotherapy (Zhang et al., 2024). Studies have found that the silence of keratin 17 (KRT17) results in significantly reduced expression of mesenchymal DNA in cancer cells leading to decreased migratory, invasive abilities, and reversing the resistance to cisplatin (Jang et al., 2022). The level of interleukin-8 (IL-8) is also found to be upregulated in a range of cancers and contribute to EMT and resistance to cisplatin in OSCC cells, while the blocking of it can reverse these effects (Joshi et al., 2023).

### 4.6 Non-coding RNA-based mechanisms

About 98% of the human genome is transcribed into RNA without the ability to code protein, which is commonly referred to as non-coding RNA (ncRNA). These ncRNAs are significantly involved in various physiological and pathological processes (Adams et al., 2017). Increasing evidence also indicates that ncRNAs can lead to cisplatin resistance in OSCC cells (Meng et al., 2021).

As a group of small ncRNAs, microRNAs (miRNAs) act as regulatory molecules in cancer, which play dual roles as both oncogenes and tumor suppressors (Wang et al., 2020).It has been discovered that the levels of *miR-214* and *miR-23a* were increased in cisplatin-resistant OSCC cells, while the level of *miR-21* was reduced. Moreover, the silencing or overexpressing of the expression level can reverse the resistance (Yu et al., 2010). It has been shown that cancer-associated fibroblast-derived extracellular vesicle (EV) treats OSCC cells to acquire cisplatin resistance, by upregulating the expression of GATA-binding protein 1 (GATA1), a downstream factor of miR-876-3p. Silencing miR-876-3p can inhibit cisplatin resistance in OSCC cells (Kang et al., 2024).

Circular ncRNAs (circRNAs) are transcripts that exhibit a closed-loop structure, which contributes to oncogenic processes (Lei et al., 2020). *Circ_0109291* and *circ-ILF2* are highly expressed in cisplatin-resistant OSCC cells compared with sensitive ones. As a sponge of *miR-188-3p*, knockdown of *circ_0109291* and overexpression of *miR-188-3p* suppressed the resistance to cisplatin by enhancing the apoptosis of the resistant tumor cells. As a negatively regulated downstream factor of *miR-188-3p*, suppressing the expression of ATP-binding cassette sub-family B member 1 (*ABCB1*) can reverse the silencing of *circ_010929*. The regulation of *circ_0109291* is a potential strategy to alleviate the cisplatin resistance of OSCC cells (Gao et al., 2022). *Circ-ILF2* serves as a sponge of *miR-1252,* downregulating the level of Krüppel-like factor 8 (*KLF8*) in OSCC cells. The upregulation of *circ-ILF2* can greatly decrease cisplatin-induced apoptosis and induce the M2 polarization of macrophages through *circ-ILF2/miR-1252*/*KLF8* axis (Wu et al., 2023b).

Plenty of long ncRNAs (lncRNAs) have also been found to play a crucial rule in chemoresistance in OSCC cells (Meng et al., 2021). Similar to circRNAs, lncRNAs can also act as sponges to impair the modulations of miRNAs on mRNAs (Thomson and Dinger, 2016). The level of lncRNA Homeobox A11 antisense RNA (HOXA11-AS) was discovered to be upregulated in OSCC cells compared to neighboring healthy human oral cells (Niu et al., 2020). The upregulation of HOXA11-AS can lower the level of *miR-214-3p*, thereby positively regulates the proto-oncogene serine/threonine-protein kinase (PIM1). The knockdown of HOXA11-AS and PIM1 or the upregulation of *miR-214-3p* can increased cisplatin-induced cytotoxicity and reverse cisplatin resistance of OSCC cells (Wang et al., 2019).

## 5 Reversing cisplatin resistance in OSCC cells by inducing ferroptosis

**FIGURE 3:**
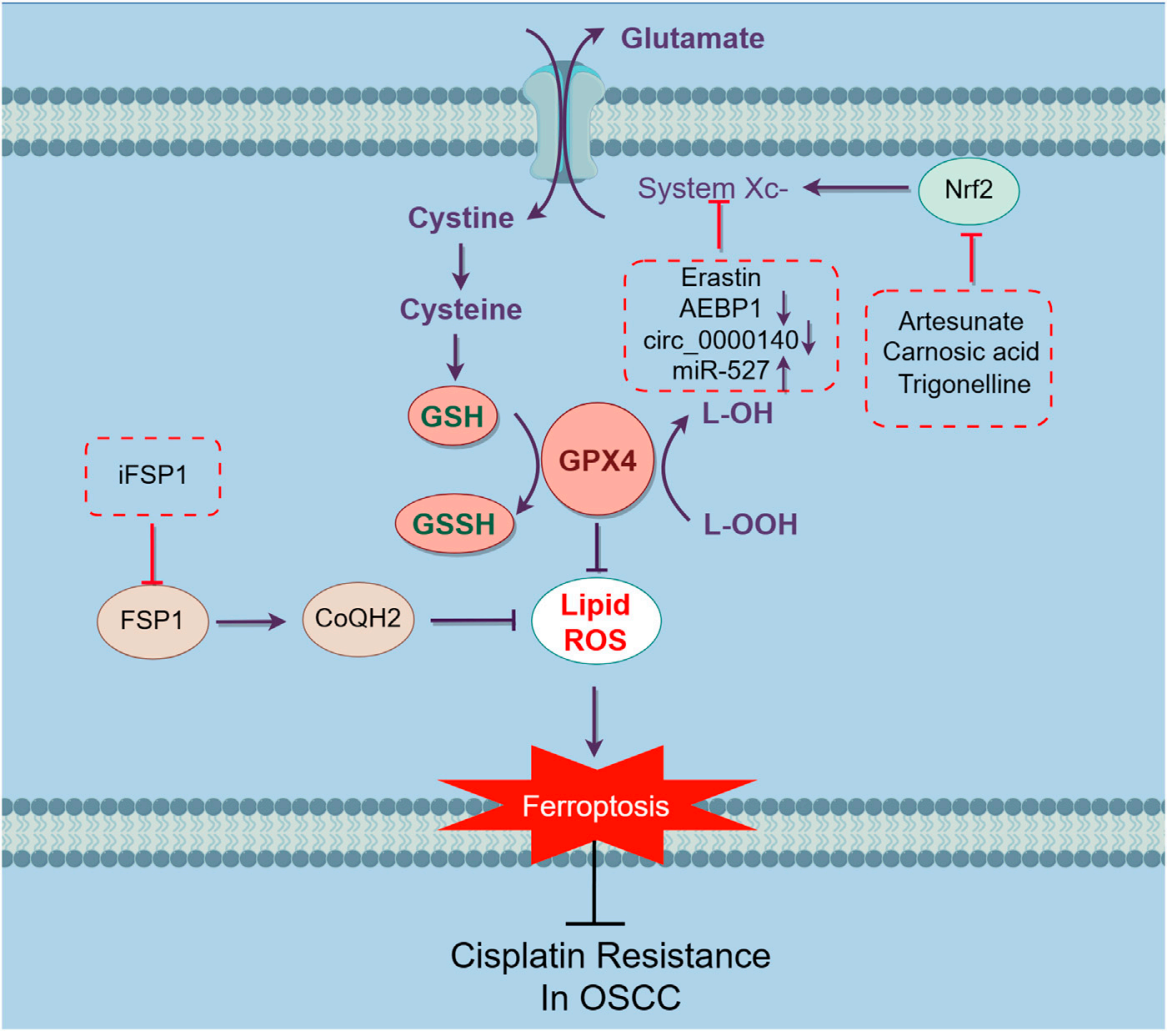
Mechanisms inducing ferroptosis and reversing cisplatin-resistance in OSCC cells. Targeting ferroptosis is an effective way to sensitize cisplatin-resistant OSCC cells. Ferroptosis is triggered by the accumulation of iron-based lipid peroxides and ROS. The main mechanism of inhibiting ferroptosis can be separated into three parts, inhibiting the build-up of Fe2^+^, suppressing the accumulation of iron dependent lipid peroxides and reducing ROS through intracellular antioxidant (Figure 1). The strategies have been discovered to overcome cisplatin-resistance in OSCC basically focused on SLC7A11/GSH/GPX4 pathway. Inhibit system Xc- by earstin or silence AEBP1 and circ_0000140, downregulate the upstream regulation factor Nrf2 through artesunate, carnosic acid and trigonelline, thereby inhibiting the SLC7A11/GSH/GPX4 pathway. Block FSP1 through iFSP1, reducing the level of CoQH2, impairing the capacity of cells to traps lipophilic free radicals. Altogether, through these ways can induce ferroptosis in cisplatin-resistant OSCC cells, thereby reversing the resistance to eliminate malignant cells.

Chemoresistance is one of the most common reasons for the failure of traditional tumor treatments. Research has shown that melanoma cells exhibit a preference for metastasizing via the lymphatic system rather than the bloodstream, which attributes to the elevated levels of GSH and the reduced availability of free iron in the lymphatic environment (Ubellacker et al., 2020), indicating that ferroptosis plays a crucial role in tumor suppression and results in the improvement of prognosis. For example, ferroptosis can interfere with lipid metabolism and disrupt iron metabolic equilibrium in chemoresistance colorectal cancer cells, thereby reversing drug resistance (Wang et al., 2023). Moreover, ferroptosis reverses the sorafenib resistance in hepatocellular carcinoma cancer cells (Guo et al., 2023) (Figure 3).

Cisplatin-resistant HNSCC cells exhibit lower level of ROS and lipid peroxidation compared to sensitive ones after being exposed to cisplatin, which are also less sensitive to ferroptosis inducers. Treating with like N-acetyl cysteine (NAC), a free radical scavenger, reverses the effect of ferroptosis inducers and cisplatin (Table 1). Indication that cisplatin-resistant HNSCC cells are also resistant to ferroptosis. Bioinformatic analysis also shows that the development of cisplatin-resistance results in gene mutations, increased gene expression associated with EMT and β-catenin signaling, as well as decreased gene expression related to the G2/M checkpoint (Yu et al., 2020). Thereby, inducing ferroptosis in these resistant cells could be a potential strategy to reverse cisplatin-resistance.

**TABLE 1:** Drugs inducing ferroptosis to reverse cisplatin resistance in OSCC and HNC cell.

Drug/Target point	Caners	Major mechanisms and outcomes	Supplementary effects
Erastin and SAS (Roh et al., 2016)	HNC	Inhibits system Xc- and conditioned media with deficient cystine	↓after DFO or Fer-1 treatment
SSZ (Zhou et al., 2022)	OSCC	Increases Fe^2+^ and ROS, inhibits GPX4 pathway, thereby limiting cell proliferation	ND
Arts and Trig (Roh et al., 2017)	HNC	Increases ROS, inhibits Nrf2 and GSH pathways, thereby inducing ferroptosis	↓after trolox, DFO or Fer-1 treatment
CA (Han et al., 2022)	OSCC	Increases ROS and lipid peroxidation, through inhibiting GSH, Nrf2 and HO-1	↓after Lip-1 treatment
circ_0000140 (Ma et al., 2023)	OSCC	Silences circ_0000140 in resistance cells, leads to deregulation of SL7A11, increased level of miR-527, ROS and Fe^2+^	ND
iFSP1 (Wu et al., 2024)	HNC	Inhibits ACSL4, GPX4, SOD2, suppress cell proliferation and tumor growth	ND

### 5.1 Blocking GPX4 pathway by system Xc-inhibitors and SSZ

Studies also reveal that inducing ferroptosis might be a latent way to reverse cisplatin resistance in OSCCs. As mentioned before, system XC-/GSH/GPX4 is a pivotal ferroptosis-inhibiting pathway. Researchers have discovered that both cisplatin-sensitive and resistant OSCC cells can induce ferroptosis in a conditioned media without cystine or with excessive glutamine while the apoptosis markers remain. This effect can be blocked by ferroptosis inducer, but not necroptosis inhibitor. After being exposed to erastin, an inhibitor of system XC-, all resistant cells significantly increased their susceptibility to cisplatin and caused ferroptosis. Moreover, similar results were achieved on nude mice models (Roh et al., 2016).

As a kind of ferroptosis inducer, sulfasalazine (SSZ) can enhance the anti-cancer effects of cisplatin by inhibiting the system Xc-in colorectal cancer cells (Ma et al., 2015). As a potential oncogene, Adipocyte enhancer-binding protein 1 (AEBP1) is upregulated in various malignant cells (Majdalawieh et al., 2020). It is shown that cisplatin-resistant OSCC cells are also less sensitive to SSZ treatment. However, AEBP1 silencing significantly sensitizes cisplatin-resistant OSCC cells to SSZ, leading to an increasing the level of Fe^2+^ and ROS, with a decreasing one with GSH, which induces ferroptosis in both vivo and vitro, thereby reversing the resistance to cisplatin (Zhou et al., 2022).

### 5.2 Attenuating the expression of Nrf2 through inhibitor and carnosic acid

Carnosic acid (CA), a polyphenolic abietane diterpene found in rosemary and common sage, has been proven to exhibit potent activity against lipid peroxidation and carcinogen detoxification enzymes in suppressing oral carcinogenesis (Manoharan et al., 2010; Das et al., 2019). Treatment with CA increases sensitivity to cisplatin in cisplatin-resistant OSCC cells by inducing ferroptosis via the blocking of the Nrf2/system XC- pathway. The expression of GSH upregulates, while ROS and lipid peroxidation downregulate in the cells, and CA can reverse the expression. Furthermore, reactivating the Nrf2 signaling and utilizing ferroptosis antagonist can counteract the suppression, indicating that inducing ferroptosis can be a potential strategy to overcome cisplatin resistance (Han et al., 2022).

Artesunate (Arts) is a semi-synthetic derivative of artemisinin, which has been found to manifest anticancer effects through inducing oxidative DNA damage and ROS production (Yu et al., 2019). It is also shown to induce ferroptosis in glioblastoma cells (Song et al., 2022). However, this capacity is limited in cisplatin-resistant HNC due to the upregulation of Nrf2 in the cells (Shin et al., 2018). Trigonelline (Trig), a Nrf2 inhibitor contributes to attenuating this resistance. Cooperating with inhibitor, Arts can increase the ability to induce ferroptosis, indicating that this resistance is partly due to Nrf2 overexpression. Combined utilization of Nrf2 inhibitor and Arts can be a promising therapeutic strategy in overcoming the cisplatin resistance in HNC cells (Roh et al., 2017). It has been discovered that combined treatment with Arts, cisplatin and iron can greatly enhance the effect of cisplatin and induce a stronger apoptosis in HNSCC cells (Okamoto et al., 2023). Similar result is also shown in myeloma cells. Arts can increase the level of Fe^2+^, ROS and ACSL4, while the expression of GPX4 decreased, thereby inducing ferroptosis, which can be reversed by ferroptosis inhibitor (Liang et al., 2023).

### 5.3 Regulation of non-coding RNA and FSP1 pathway

Altered expression patterns of circRNAs have been discovered in OSCC (Paramasivam, 2022). It should be noted that certain circRNAs relate to cisplatin resistance in OSCC cells (Qiu et al., 2021; Gao et al., 2022). Moreover, the overexpression of *circ_0000140* was discovered in cisplatin-resistant OSCC cell lines. Interestingly, knocking down this circRNA remarkably reverses the cisplatin resistance and promotes ferroptosis. The overexpression of *circ_0000140* results in the dysregulation of the *miR-527*, thereby affecting the expression of SLC7A11 to inhibit ferroptosis, and the upregulation of *miR-527* and the downregulation of SLC7A11 can reverse this effect (Ma et al., 2023).

Drug-tolerant persister (DTP) is a prestate of drug-resistant cells, which enables malignant cells to endure the chemotherapy for a period, thereby allowing them to develop drug resistance through different methods (Cabanos and Hata, 2021). As a parallel system to the system Xc-/GSH/GPX4 pathway, FSP1 generates an antioxidant form of CoQH_2_ thereby inhibiting ferroptosis. Interestingly, FSP1 is highly expressed in HNC cells from cisplatin-resistant patients. The overexpression of FSP1 is also discovered in DTP cells which results in cisplatin resistance, enhanced migratory and invasive capacities. Not only can the FSP1 inhibitor, iFSP1, and shRNA reverse the capacity, but allow cisplatin to exhibit a more potent effect (Wu et al., 2024).

## 6 Conclusion

In brief, ferroptosis might be a potential strategy to overcome cisplatin resistance in OSCC cells. Combined treatment with ferroptosis inducers and chemotherapy could be a potent way to increase the 5-year survival rate and the prognosis of OSCC patients. However, the underlying mechanisms of how ferroptosis reverses cisplatin resistance still remains unclear, making it still far away from clinical practice of drug combination of cisplatin and ferroptosis inducers. Furthermore, ferroptosis can aggravate inflammation in a variety of disease. Treatment with ferroptosis inducers would be harmful to those cancer patients with underlying conditions. It might be benefit to combine with targeted treating, through directly targeting malignant cells, alleviating or avoiding unnecessary damage to healthy cells.

Nowadays, bioinformatic analysis has been widely used to predict the possible function of various genes and protein in cancers, thereby screening targeting points. Further research could focus on these bioinformatic analysis provided biomarkers, predicting the efficacy of ferroptosis inducers in OSCC, exploring new ferroptosis regulators, understanding the complicated molecule mechanisms, refining therapeutic procedures, and providing personalized medicine strategies. It is believed that in the near future, researchers will provide more combined treatment strategies to eliminate malignant cells.
